# Discovering Biomarkers for Non-Alcoholic Steatohepatitis Patients with and without Hepatocellular Carcinoma Using Fecal Metaproteomics

**DOI:** 10.3390/ijms23168841

**Published:** 2022-08-09

**Authors:** Svenja Sydor, Christian Dandyk, Johannes Schwerdt, Paul Manka, Dirk Benndorf, Theresa Lehmann, Kay Schallert, Maximilian Wolf, Udo Reichl, Ali Canbay, Lars P. Bechmann, Robert Heyer

**Affiliations:** 1 Department of Internal Medicine, University Hospital Knappschaftskrankenhaus, Ruhr-University Bochum, In der Schornau 23–25, 44892 Bochum, Germany; 2 Bioprocess Engineering, Otto von Guericke University Magdeburg, Universitätsplatz 2, 39106 Magdeburg, Germany; 3 Data and Knowledge Engineering Group, Otto von Guericke University Magdeburg, Universitätsplatz 2, 39106 Magdeburg, Germany; 4 Max Planck Institute for Dynamics of Complex Technical Systems, Bioprocess Engineering, Otto von Guericke University Magdeburg, Sandtorstraße 1, 39106 Magdeburg, Germany; 5 Multidimensional Omics Analysis Group, Leibniz-Institut für Analytische Wissenschaften—ISAS—e.V., Bunsen-Kirchhoff-Straße 11, 44139 Dortmund, Germany; 6 Faculty of Technology, Bielefeld University, Universitätsstraße 25, 33615 Bielefeld, Germany

**Keywords:** metaproteomics, fecal microbiota, non-alcoholic steatohepatitis, hepatocellular carcinoma, machine learning

## Abstract

High-calorie diets lead to hepatic steatosis and to the development of non-alcoholic fatty liver disease (NAFLD), which can evolve over many years into the inflammatory form of non-alcoholic steatohepatitis (NASH), posing a risk for the development of hepatocellular carcinoma (HCC). Due to diet and liver alteration, the axis between liver and gut is disturbed, resulting in gut microbiome alterations. Consequently, detecting these gut microbiome alterations represents a promising strategy for early NASH and HCC detection. We analyzed medical parameters and the fecal metaproteome of 19 healthy controls, 32 NASH patients, and 29 HCC patients, targeting the discovery of diagnostic biomarkers. Here, NASH and HCC resulted in increased inflammation status and shifts within the composition of the gut microbiome. An increased abundance of kielin/chordin, E3 ubiquitin ligase, and nucleophosmin 1 represented valuable fecal biomarkers, indicating disease-related changes in the liver. Although a single biomarker failed to separate NASH and HCC, machine learning-based classification algorithms provided an 86% accuracy in distinguishing between controls, NASH, and HCC. Fecal metaproteomics enables early detection of NASH and HCC by providing single biomarkers and machine learning-based metaprotein panels.

## 1. Introduction

During the last few decades, the prevalence of obesity and metabolic syndrome has increased tremendously [1]. Due to this, non-alcoholic fatty liver disease (NAFLD) has emerged as one of the leading causes of chronic liver diseases worldwide [2]. The NAFLD spectrum ranges from visible fatty degeneration of the organ, but can progress to the inflammatory form of non-alcoholic steatohepatitis (NASH) [3,4]. Due to local and systemic inflammatory processes, this chronic inflammation carries the risk of further disease progression and cancer development [5]. Different mechanisms related to fat and glucose metabolism can promote fibrotic remodeling of the organ, the development of cirrhosis, and even hepatocellular carcinoma (HCC), whereas HCC is one of the most common cancers and causes of cancer-associated deaths worldwide [6,7]. To date, not all mechanisms affecting the progression of NAFLD have been elucidated. For example, fibrosis is a main driver of HCC development, whereas NASH can develop into HCC even without prior cirrhosis [8].

Timely diagnosis and progress prediction of NAFLD, NASH, and HCC is a major challenge, as it remains clinically inconspicuous for long periods and lacks appropriate biomarkers applicable for preventive medical checkups. Several factors and mechanisms that affect NAFLD’s progress are currently discussed, such as the presence of diabetes and metabolic syndrome, and the composition of intestinal microbiota [9]. We and others described the interaction between the gut and liver diseases within the enterohepatic circulation. Shifts in the composition of the intestinal microbiome affect bile acid composition and the formation of bioactive metabolites and substrates that can affect fatty acid and glucose metabolism, and thus, also promote NAFLD and its progress [10,11,12,13,14]. Based on sequencing methods, gut microbiota compositions have previously been described in different measures and NAFLD patient cohorts. However, most findings were descriptive, and the underlying mechanisms have not been fully elucidated. In contrast to monitoring the taxonomic composition by sequencing methods, metaproteomics detects the actual gene expression. Furthermore, fecal metaproteomics also reveals host proteins (e.g., from the immune system), indicating health status [15] or problems with food digestion [16].

Consequently, metaproteomics is a promising method for preventive, non-invasive medical checkups, but we still lack meaningful biomarkers. To identify the required biomarkers and to understand the correlation between the pathogenesis and the gut microbiome in NASH and NASH-derived HCC, we analyzed the human proteins and the taxonomic and functional gut microbiome composition through fecal metaproteomics.

## 2. Results

### 2.1. Characteristics of the Study Cohort

Eighty subjects including healthy controls (n = 19, 23.5 average age, and 23.0 BMI), NASH patients (n = 32, 53.3 average age, and 30.9 BMI), and HCC patients (n = 29, 67.8 average age, and 30.3 BMI), were included. The increasing age and BMI from healthy controls to NASH and HCC patients reflected the progression of disease over time due to elevated BMI. Demographic data of transient elastography (TE) and different serum parameters of individual patient groups are depicted in Supplementary Table S1.

Patients (NASH and HCC) showed significantly increased liver stiffness and hepatic fat accumulation compared to controls, as assessed by TE, including measurement of the controlled attenuation parameter (CAP). Tumor markers such as alpha-fetoprotein-Centaur (AFP), lectin-3-reactive alpha-fetoprotein (AFP-L3), and des-gamma-carboxyprothrombin (DCP) also showed a slight increase in NASH; however, this increase was below the established cutoff values for tumor diagnosis in NASH, but highly increased in HCC. Furthermore, NASH and HCC patients possessed elevated serum parameters of liver injury such as alanine aminotransferase (ALT), aspartate aminotransferase (AST), alkaline phosphatase (AP), gamma-glutamyltransferase (γGT), glutamate dehydrogenase (GLDH), and lactate dehydrogenase (LDH). Serum levels of bilirubin were not changed when comparing the groups.

Total bile acids and their target, fibroblast growth factor 19 (FGF19), were increased in the serum of NASH and were even higher in HCC. Regarding the triglyceride (TAG) levels, a significantly higher concentration could be observed for NASH and HCC. Although not significantly, in NASH and HCC patients, low-density lipoprotein (LDL) cholesterol was increased, whereas high-density lipoprotein (HDL) cholesterol levels showed a significant decrease. As important metabolic mediators, we measured serum levels of adiponectin, glucagon-like peptide-1 (GLP1), and FGF21. GLP1 and FGF21 levels were significantly increased in NASH and HCC compared to controls, whereas adiponectin levels were elevated but without statistical significance. Cell death marker M65, apoptosis marker M30, and serum levels of pro-inflammatory cytokines interleukin 6 (IL6) and tumor necrosis factor alpha (TNFα) were significantly increased in NASH and HCC. This increase was accompanied by higher levels of the inflammation-associated protein lipoprotein-binding protein 1 (LBP1) (Supplementary Table S1).

### 2.2. Characterization of Fecal Metaproteomics

The metaproteomic analysis yielded an average of 19,221 ± 6752 identified spectra for each patient, which were assigned, on average, to 4218 ± 940 metaproteins and 193 ± 20 taxonomic families (Supplementary Tables S2 and S3). The taxonomic assignment of all identified spectra (Supplementary Figure S1) resulted in an average of 11.41 ± 2.51% bacterial spectra, 0.71 ± 0.20% archaeal spectra, 5.41 ± 1.79% eukaryotic spectra, and 0.32 ± 0.10% viral spectra. Furthermore, 30% of the identified spectra belonged to unknown protein entries from the metagenome, and for 36.5%, no specific superkingdom could be assigned due to overlapping protein identifications.

The set of eukaryotic spectra was divided into a fraction belonging to the host (Hominidae: 1.78 ± 1.01%) and a fraction belonging to photosynthetically active eukaryotes related to diet (e.g., Poaceae: 0.39 ± 0.27%). However, most eukaryotic spectra lack a sufficiently precise taxonomic assignment to assign them to one of these two groups without detailed functional interpretation. The most important microbial families were Bacillaceae, 2.01 ± 0.55%; Enterobacteriaceae, 1.70 ± 0.42%; Clostridiaceae, 0.84 ± 0.25%; Mycobacteriaceae, 0.40 ± 0.14%; and Pasteurellaceae, 0.37 ± 0.12%.

The main metabolic functions were summarized based on Biemann et al. 2021 [16], comprising human and microbial hydrolysis, microbial metabolisms and transporters, and host proteins derived from the intestinal barrier and the immune system (Supplementary Table S2).

### 2.3. Identification of Disease-Specific Metaprotein Patterns

**Figure 1:**
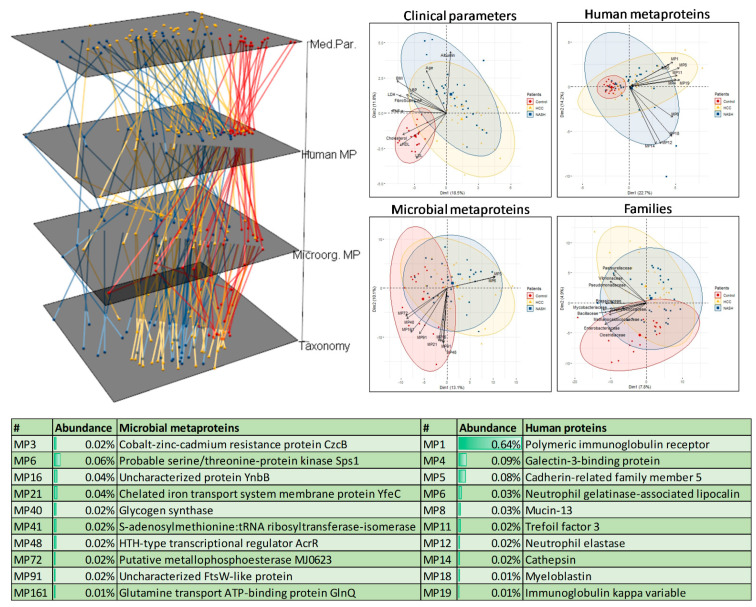
Multilayer PCA of clinical parameters, human and metaproteins, and family-level taxonomy for all samples. The individual PCAs were visualized on the left, and same samples were connected across all layers. The associated biplots with the top 10 loadings were shown on the right side. For human and microbial metaproteins, the metaproteins with at least 0.01% of the total spectral count were selected. In contrast, for families and clinical parameters, all 419 and 30 were chosen, respectively. For better readability, the top ten human and microbial loadings (metaproteins) were summarized in a table below the plot.

To identify possible disease-specific patterns, we compared the human (family *Hominidae*) and bacterial metaproteins with the measured medical parameters and taxonomic composition at the family level using multilayer principal component analysis (PCA) (Figure 1) and an analysis of similarities (ANOSIM) (Supplementary Table S4).

The medical parameters allowed a separation of the healthy and diseased people, matching weak (0.12 < R < 0.20, *p*-value < 0.01) but significant differences found by ANOSIM for all three groups. Based on microbial metaproteins and families, only a separation between controls and the diseased was possible by the ANOSIM, and a weak trend in the PCAs was observable (Figure 1). In contrast, the ANOSIM of the human proteins enabled no significant differences between the three groups (*p*-values > 0.26). However, the human metaprotein profiles of the healthy individuals were closer together than those of the diseased patients.

Interlayer connections between the PCAs showed that the control samples varied less than the diseased samples across all layers. Analysis of the PCA loadings showed that NASH and HCC were correlated, among others, with increased blood fat levels, albumin levels, age, and a lower abundance of the families *Clostridiaceae* and *Enterobacteriaceae* (Figure 1). Furthermore, NASH and HCC patients’ feces contained more antibodies and metaproteins associated with the gut barrier and immune system (e.g., polymeric immunoglobulin receptor). Regarding microbial metaproteins, correlations with low-abundant metaproteins such as MP6 and a probable serine/threonine protein kinase, SPs1, were observable.

### 2.4. Significantly Altered Metaproteins, Taxonomies, and Functions

To identify potential diagnostic NASH and HCC biomarkers, we considered all metabolic functions from our summary and all metaproteins and families whose identified spectra abundance was above 0.01% (Supplementary Tables S3 and S5). In total, 15 of 40 functions and 9 of 34 families were significantly altered (*p*-value < 0.01). For the metaproteins, we increased the significance threshold to *p* < 10^−5^, revealing 25 of 961 changed metaproteins.

**Figure 2:**
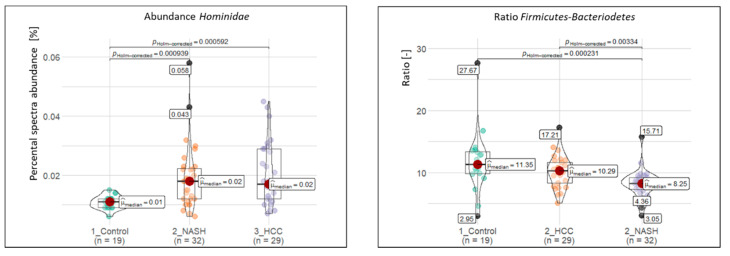
Abundance of the family Hominidae and the ratio of Bacteriodetes to Firmicutes. The abundance is based on the normalized abundance of identified spectra.

**Figure 3:**
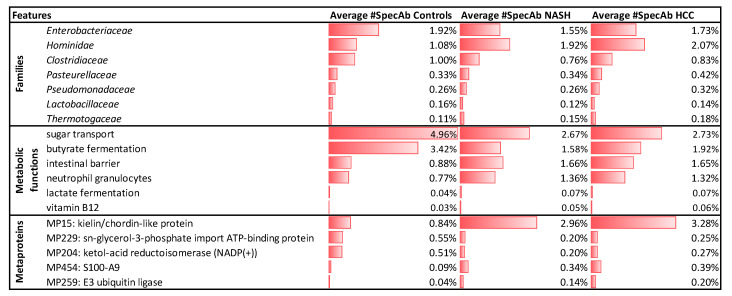
Summary of selected changed features. Significance was evaluated by the Kruskal–Wallis test using a *p*-value cutoff smaller than 0.01 for families and metabolic functions and smaller than 10^−5^ for metaproteins.

Among others, we observed in NASH and HCC patients more proteins assigned to *Hominidae* (spectral abundance (#SpecAb)__C_: 1.08%, #SpecAb__NASH_: 1.92%, #SpecAb__HCC_: 2.07%) (Figure 2) and *Thermotogaceae* (#SpecAb__C_: 0.11%, #SpecAb__NASH_: 0.15%, #SpecAb__HCC_: 0.18%; Figure 3), and less assigned to *Enterobacteriaceae* (#SpecAb__C_: 1.92%, #SpecAb__NASH_: 1.55%, #SpecAb__HCC_: 1.73%), *Clostridiaceae* (#SpecAb__C_: 1.00%, #SpecAb__NASH_: 0.76%, #SpecAb__HCC_: 0.83%), and *Lactobacillaceae* (#SpecAb__C_: 0.16%, #SpecAb__NASH_: 0.12%, #SpecAb__HCC_: 0.14%) (Figure 3).

Furthermore, in NASH and HCC patients more proteins for the intestinal barrier (#SpecAb__C_: 0.88%, #SpecAb__NASH_: 1.66%, #SpecAb__HCC_: 1.65%) and neutrophil granulocytes were detected (#SpecAb__C_: 0.77%, #SpecAb__NASH_: 1.36%, #SpecAb__HCC_: 1.32%). Within the microbiome, we observed decreased microbial metabolism (e.g., butyrate fermentation; #SpecAb__C_: 3.42%, #SpecAb__NASH_: 1.58%, #SpecAb__HCC_: 1.92%) and transporters (e.g., sugar transport; #SpecAb__C_: 4.96%, #SpecAb__NASH_: 2.67%, #SpecAb__HCC_: 2.73%) in NASH and HCC patients. Exceptions were transporters for vitamin B12 (#SpecAb__C_: 0.03%, #SpecAb__NASH_: 0.05%, #SpecAb__HCC_: 0.06%) and lactate fermentation (#SpecAb__C_: 0.04%, #SpecAb__NASH_: 0.07%, #SpecAb__HCC_: 0.07%), being more abundant in NASH and HCC patients. Potential marker metaproteins for NASH and HCC were a decreased abundance of the sn-glycerol-3-phosphate import ATP-binding protein (#SpecAb__C_: 0.55%%, #SpecAb__NASH_: 0.20%, #SpecAb__HCC_: 0.25%; unknown superkingdom) and ketol-acid reductoisomerase (NADP(+)) (#SpecAb__C_: 0.51%, #SpecAb__NASH_: 0.20%, #SpecAb__HCC_: 0.27%), and increased abundances for the kielin/chordin-like protein (#SpecAb__C_: 0.84%, #SpecAb__NASH_: 2.96%, #SpecAb__HCC_: 3.28%; class: *Mammalia*) and protein S100-A9 (#SpecAb__C_: 0.09%, #SpecAb__NASH_: 0.34%, #SpecAb__HCC_: 0.39%; unknown superkingdom). Furthermore, we observed in NASH and HCC patients an increased abundance of the E3 ubiquitin ligase (#SpecAb__C_: 0.04%, #SpecAb__NASH_: 0.14%, #SpecAb__HCC_: 0.20%) (Figure 3). Although this metaprotein was assigned to the fungal species *Arthroderma otae,* we would speculate that it belongs actually to the host, since it was much more abundant than other low-abundant metaproteins assigned to fungi.

Unfortunately, identified markers only enabled to separate between controls and diseased people, but not between NASH and HCC. The only exceptions were an increased abundance of *Pasteurellaceae* (#SpecAb__C_: 0.33%, #SpecAb__NASH_: 0.34%, #SpecAb__HCC_: 0.42%%) and *Pseudomonadaceae* (#SpecAb__C_: 0.26%%, #SpecAb__NASH_: 0.26%%, #SpecAb__HCC_: 0.32%%) in the feces of HCC patients and a decreased ratio between *Firmicutes* and *Bacteriodetes* in NASH samples (Figure 2).

### 2.5. Potential Biomarkers to Distinguish NASH and HCC from Controls

In the next step, we evaluated the performance of the significantly changed top ten metaproteins for separating between healthy and diseased patients (Supplementary Table S5). Therefore, we performed an ROC curve analysis and compared the area under the curve (Table 1). For the analyzed metaproteins, the area varied between 0.913 and 0.815, indicating a good classification. Exemplary, for the kielin/chordin-like protein, about 80% of the diseased people could be diagnosed with only 10% false positives. However, the number of false positives was still too high for routine diagnosis or preventive medical checkups.

### 2.6. Machine Learning-Based Biomarker Panels to Separate NASH from HCC and Controls

**Figure 4:**
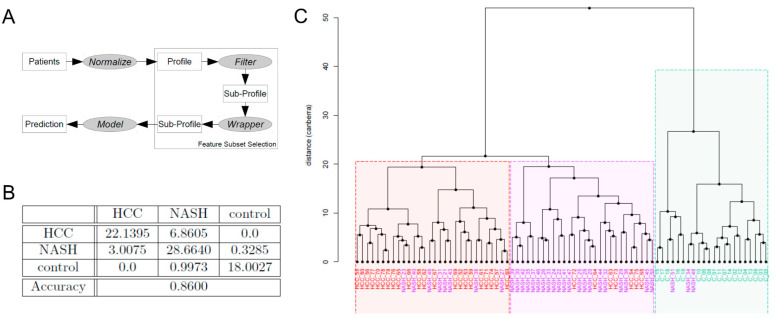
Machine learning-based sample classification between NASH, HCC, and controls. (**A**) shows the workflow of the software for feature selection, feature wrapping, and development of the classification algorithms. (**B**) shows display of the confusion matrix of the best-observed classifier (linear discriminant analysis.). Cross-validation ensured that the patient numbers in the confusion matrix were real and not natural numbers. The evaluation was performed by averaging a 5-fold cross-validation on 10,000 repeats. (**C**) shows the clustering of the data and their intrinsic similarity using Ward linkage and Canberra distances.

To improve the diagnosis of NASH and HCC, we developed machine learning-based classification algorithms (Figure 4A). Therefore, we ranked the normalized features according to their *p*-values derived from a *t*-test. Subsequently, we applied the wrapper technique on the top-ranked features to further reduce the set to the most relevant molecules for the classification task. Under several models, the diagonal linear discriminant analysis and logistic regression performed best and enabled a separation of controls from NASH or HCC at 99.98% and 100% using seven and five features, respectively. In contrast, the correct distinction between NASH and HCC was only possible in 86.4% of samples using ten features. Consequently, the correct classification of all three groups was only possible in 86.0% of all cases using eleven features (Table 2). Thereby, the NASH samples were either wrongly classified as healthy or HCC, and HCC patients were wrongly classified as NASH patients (Figure 4C). Misclassification of HCC as controls or other was not observed. Evaluating all selected features manually (Supplementary Table S6) revealed nucleophosmin as a promising biomarker. We observed a considerable overlap of identified molecules between both the machine learning and ROC curve analysis approaches, as described in Section 2.5. Ergo, both approaches complement each other in evidence. It was enriched in NASH and HCC compared to controls by a factor of 103, or rather, 129 (#SpecAb__C_: 0.00%, #SpecAb__NASH_: 0.026%, #SpecAb__HCC_: 0.033%).

## 3. Discussion

NASH and HCC are severe liver diseases that progressively reduce liver function as the disease progresses, as observed through worsened liver function parameters, liver fibrosis, and liver damage [17]. The progressive carcinogenesis in HCC patients coincided with the increased tumor markers such as AFP-Centaur, AFP-L3, and DCP [18]. A primary cause of NASH and HCC is an unhealthy diet, which is reflected in a higher BMI, worse blood lipids, and sugar parameters. The unhealthy lifestyle leads over time, as observable with the higher age of the NASH and HCC patients, to fibrosis and tumor formation [19]. NASH- and HCC-induced liver alterations indeed result in changed bile acid production and liver protein expression, leading to changes in the gut microbiome via bile acid secretion from the gallbladder. Therefore, gut microbiome alterations may indicate NASH or HCC, or could even promote it via bile acid conjugation or ethanol production [20].

Although there was no clear separation of the metaproteome of NASH and HCC patients from the controls, differences in the metaprotein and family fingerprint were already observable in the PCA plots. Thereby, differences between healthy and diseased patients were bigger than between NASH and HCC, reflecting that HCC often develops from NASH [21,22]. The bigger variations in the PCA plots between the NASH and HCC patients than within the controls show how diseases possess different forms and severities.

Subsequent identification of potential marker functions, families, and metaproteins revealed multiple potential biomarkers enabling the separation between healthy and diseased patients. However, as the ROC plots and comparison of the area under the curve showed, the biomarker accuracy was insufficient for separating NASH from HCC. A better performance provided machine learning-based classification using five to seven metaproteins. Therefore, developing comprehensive clinical panels for the fecal metaproteome similar to a blood picture may represent a promising approach for NASH and HCC diagnosis. The particular advantage of fecal metaproteomics is that samples can be taken non-invasively at home and sent to clinical laboratories, making it a perfect approach for preventive clinical checkups.

The overlap between the presented top PCA loadings, significantly altered metaproteins, taxonomies, functions, and machine learning-based features appeared small. However, the latter groups were very similar since they were based on significantly changed metaproteins, but we focused within this manuscript on different aspects. We applied a smaller *p*-value cutoff for the manual selection and concentrated on high-abundant marker proteins with a potential clinical significance. For machine learning, the algorithms selected the smallest number of features, enabling the best separation of the groups. Furthermore, evaluation of the PCA loadings revealed that most loadings also differed significantly between controls, NASH, and HCC, but not all. This observation reflects that the PCA visualizes the variance of the samples, and the top loadings indicate the main differences. However, as observable in the PCA plots, NASH, HCC, and control samples were only weakly separated, also suggesting other differences within the microbiome.

Although we identified several promising marker metaproteins in our cohort, they required a clinical evaluation since they could also be linked with other diseases or with lifestyle. Elevated levels of kielin/chordin, an E3 ubiquitin ligase, and nucleophosmin 1 could be directly involved in the pathogenesis. NASH results in the accumulation of adipose cells in the liver, secreting tumor growth factor (TGFβ). TGFβ indeed is induced via endoplasmic reticulum (ER) stress and the unfolded protein response, apoptosis, and thus, fibrosis [23,24,25]. The E3 ubiquitin ligase is involved in protein degradation and nucleophosmin 1 in nucleic transport and ribosome biosynthesis. Upregulation of both would fit to enhance the unfolded protein response. Furthermore, both are described as enhanced in liver cancers [25,26]. Kielin/chordin indeed represses TGFß signaling, representing a protection mechanism against NASH. Soofi et al. showed that kielin/chordin knockout mice were more susceptible to developing hepatic steatosis and liver fibrosis [27]. Conversely, overexpression of kielin/chordin protected the mice’s liver from the effects of an excessively high-fat diet.

In contrast to these three disease-specific biomarkers, increased abundance of the family *Hominidae* combined with more metaproteins derived from the immune system (e.g., calprotectin from neutrophilic granulocytes) and the gut barrier reflected a worsened health status of NASH and HCC patients in contrast to the healthy control. However, this is not specific to NASH or HCC. For example, Lehmann et al. [15] observed higher abundances of calprotectin (protein S100-A9) in the feces of patients with inflammatory bowel disease, and Biemann et al. (2021) [16] showed that obese patients possess a systemic inflammation, higher abundances of protein S100-A9, and increased abundance of the family *Thermotogaceae*.

Similarly, obesity and diet also explain gut microbiome alterations, including decreased microbial transporters or less butyrate fermentation. Increased food uptake elevates the production and secretion of bile acids into the gut. Bile acids indeed possess antimicrobial properties, leading to an altered gut microbiome [28]. Although obesity is linked with an increased abundance of nutrients, the observed increase in vitamin B12 transporters suggests a higher competition for vitamins and a potential lack. In line with this, Voland et al. (2021) [29] reviewed the lack of vitamins in obese people and its impact on the gut microbiome, which could also explain the altered ratio of *Firmicutes* to *Bacteriodetes* in NASH patients [30]. On the contrary, there are also some research articles suggesting that the gut microbiome contributes to NASH and HCC by the production of toxic components such as alcohol, toxic bile acids, or inflammatory microbial metabolites and the activation of cancerogenesis-associated signaling pathways [10,31,32,33]. We observed some hints, such as an increase in NASH and HCC patients of probable serine/threonine protein kinase SPs1, which is associated with signaling [34], and of pyruvate decarboxylase isozyme 3 for ethanol production [35]. However, the evidence was insufficient, or the alteration was not significant.

The main shortcoming of the study was that no patient’s specific metagenomes were available. Therefore, and to keep this study comparable with previous studies [15,16], we selected the same metagenome database. However, specific metagenomes would increase the number of identified metaproteins, the taxonomic and functional protein annotation, and the protein grouping. Since our study focused on identifying fecal marker proteins, we included no liver or epithelial biopsies. Therefore, spatial protein assignment was impossible, but would be valuable for mechanistic studies. For example, the identified polymeric immunoglobulin receptor is upregulated in the liver of patients with liver fibrosis and liver cancer [36,37]. However, the observed increase is more likely caused by the degradation of the intestinal epithelia expressing the receptor in high amounts.

Analogous to all other NASH and HCC studies, our study suffers from the dependency of NASH and HCC on the cofounding factors of lifestyle, age, and co-morbidity with other diseases. For example, NASH is caused by a calorie-rich diet and is a metabolic syndrome. Thus, it often occurs together with diabetes and hypertension. In future studies, documentation of dietary habits of the patients would be useful since diet may influence the composition of the gut microbiota and may have an impact on the development of diseases, especially cancer [38].

## 4. Conclusions

In conclusion, we proposed, by fecal metaproteomics, several potential biomarkers enabling the separation of NASH and HCC patients from healthy people, presenting a valuable tool for preventive medical checkups. An even better diagnosis than single marker proteins, our findings provide machine learning-based biomarker panels.

## 5. Materials and Methods

### 5.1. Patient Recruitment and Sample Collection

This study was conducted based on a previous study by Sydor et al. (2020), analyzing potential links between the liver and the gut in NASH-related hepatocarcinogenesis. Therefore, they compared alterations of gut microbiota and mediators of bile acid signaling in the absence or presence of cirrhosis through analysis of feces and serum from patients with NASH and NASH-HCC and healthy volunteers [14].

The Ethics Committee (Institutional Review Board) of the University Hospital Essen (reference number: 14-6044-BO) approved the study, and all subjects provided informed written consent. The study protocol conformed to the ethical guidelines of the Declaration of Helsinki.

For the analysis, serum and fecal samples of subjects with NASH (n = 32), HCC (n = 29), and healthy controls (n = 19) without evidence of NAFLD were analyzed. The inclusion criteria for the study were the presence of NAFLD, specifically with appropriately confirmed NASH, and HCC based on NASH. Known chronic viral, toxic, hereditary, and immunologic liver diseases were considered exclusion criteria (e.g., HBV, HCV, autoimmune hepatitis, primary and secondary biliary cholangitis (PBC/PSC), Wilson’s disease, etc.).

Confounding factors of our study were age and BMI, which could not be mitigated since we wanted to focus on the development of NASH and HCC over time due to increased calorie uptake.

Diagnosis of NASH and HCC was performed as described before [14]. Patients with significant alcohol intake, as defined as consuming more than two standard drinks daily or more than six daily drinks on weekends for at least five years [39], were not considered for the study. The presence of HBV and HCV was excluded by seronegativity for HBV or HCV following standard laboratory tests. Healthy volunteers with a BMI below 30 and without NAFLD were selected as healthy controls.

All serum samples were collected in a fasted state in the morning and stored at −80 °C until measurement. The central laboratory of the University Hospital Essen evaluated by routine diagnostics the general clinical parameters, enzymes (ALT, AST, AP, γGT), total bile acids, and tumor markers (AFP, AFP-L3, and DCP). Fecal samples were collected from every patient in sterile tubes and stored at −80 °C.

### 5.2. Transient Elastography and Controlled Attenuation Parameter

Liver stiffness and the controlled attenuation parameter (CAP) to assess hepatic fat accumulation were measured using the Fibroscan^®^ (Echosens, Paris, France), with samples taken from the subjects in a fasted state.

### 5.3. ELISA

Commercially available kits were used to measure serum levels of the overall cell death marker M65 and apoptosis marker M30 (TecoMedical, Sissach, Switzerland). Quantification of serum concentrations of FGF19, FGF21, GLP1, IL6, and TNFα was performed using the specific Quantikine ELISA Kit from R&D Systems (Minneapolis, MN, USA). Serum amounts of LBP1 were quantified using the LBP ELISA Kit (Hycult Biotech Uden, Uden, The Netherlands). All procedures were performed following the manufacturer’s instructions.

### 5.4. Fecal Sample Preparation for Metaproteomics

Proteins from approx. 100–200 mg stool samples were extracted by cell lysis and phenol extraction as described in Lehmann et al. [15]. After FASP digestion [40], LC-MS/MS analysis was performed using an UltiMate 3000 RSLCnano splitless liquid chromatography system coupled online to an Orbitrap Elite ™ hybrid ion trap, the Orbitrap-MS (both from Thermo Fisher Scientific, Bremen, Germany) using a 120 min gradient. All chemicals used were at least analysis quality and the solvents used were LC-MS/MS quality.

### 5.5. Data Handling

The MetaProteomeAnalyzer (version 3.1) [40] was used for protein identification, which included the search engines X! Tandem, OMSSA, and Mascot and the following parameters: enzyme trypsin, one missed cleavage, monoisotopic mass, carbamidomethylation (cysteine) as a fixed modification, oxidation (methionine) as a variable modification, ±10 ppm precursor and ±0.5 Da MS/MS fragment tolerance, 1 13 C, +2/+3 charged peptide ions, and a false detection rate of 1%. The used protein database was the UniProtKB/Swiss-Prot database (as of 16 January 2019) combined with a human gut microbiome database [41]. A BLAST search (NCBI-Blast version 2.2.31) against UniProtKB/Swiss-Prot was carried out for proteins that could not be annotated taxonomically or functionally. All BLAST hits with the best E-value that were at least below 10^−4^ were combined and used to annotate the protein identifications. Redundant homologous protein identifications were combined into a protein group (also referred to as metaprotein) if they had at least one peptide identification in common. Finally, all results were uploaded to PRIDE (Accession: PXD034175).

### 5.6. Statistical Analysis

Statistical analysis, including the multilayer PCA, ANOSIM, Kruskal–Wallis test, and violin plots [42], was carried out using R Statistics (version 1.2.5001) and Rstudio. For Krona plots, the provided Excel template by Ondov et al. [43], and for the ROC plots, the web service from Eng et al. (2014) [44] were used. Power analysis for metaproteomics using standard deviations of three spectra from a previous study [15] showed that for 20 samples per group and for proteins with an abundance of at least five spectra, a doubling of the spectra could be observed with a power of 0.993 and with a significance value below 0.01.

### 5.7. Development of a Biomarker Panel

A comprehensive software package using R and Java was used to develop machine learning-based classification algorithms. The software ranked the normalized features according to their *t*-test-based predictive power. We considered only features when 2/3 of the samples in at least one group had measurements (values above zero). Subsequently, different feature sets were identified by the wrapper method. The following machine learning models were used: linear discriminant analysis (LDA) [45], diagonal LDA [46], logistic regression [47], support-vector machine [48], random forest [49], extremely randomized trees [50], and k-nearest neighbors [51]. The evaluation was performed by averaging a 5-fold cross-validation on 100 repeats for the tree-like models and 10,000 repeats for the rest. Results were summarized in a confusion matrix and a clustergram using Ward linkage and Canberra distances.

## Figures and Tables

**Table 1:** Potential biomarker metaproteins between controls and diseased patients. We analyzed the ROC plot analysis for the most abundant ten metaproteins and summarized the area under the curve to evaluate metaprotein biomarkers.

Metaproteins	#SpecAb	Area under Curve
*Kielin/chordin-like protein*	*2.568%*	*0.893*
*Sn-glycerol-3-phosphate import ATP-binding protein*	*0.303%*	*0.868*
*Ketol-acid reductoisomerase (NADP(+))*	*0.297%*	*0.862*
*Protein S100-A9*	*0.296%*	*0.815*
*Probable E3 ubiquitin ligase complex SCF*	*0.135%*	*0.839*
*30S ribosomal protein S3*	*0.120%*	*0.879*
*Formate-tetrahydrofolate ligase 2*	*0.073%*	*0.913*
*30S ribosomal protein S2*	*0.066%*	*0.842*
*Acyl-CoA dehydrogenase, short-chain specific*	*0.066%*	*0.883*
*Glyceraldehyde-3-phosphate dehydrogenate*	*0.063%*	*0.905*

**Table 2:** Classification accuracy for NASH, HCC, and controls. Results were obtained by the given number of features and the algorithms by averaging a 5-fold cross-validation on 10,000 repeats.

Comparison	Accuracy	Number of Features
NASH vs. Control	0.9998	7 features
HCC vs. Control:	1	5 features
HCC vs. NASH	0.8640	10 features
HCC vs. NASH vs. Control	0.86	11 features

## Data Availability

The datasets analyzed during the current study are accessible in the PRIDE Archive (Proteomics Identifications Database) under the accession number PXD034175. For reasons of data protection, patient data are presented in summarized form, but can be requested from the authors by personal inquiry.

## Supplementary Materials

The supporting information can be downloaded at: https://www.mdpi.com/article/10.3390/ijms23168841/s1.

## Abbreviations

AFP/AFP-L3	alpha-fetoprotein/lectin-3-reactive alpha-fetoprotein
ALT	alanine aminotransferase
ANOSIM	analysis of similarities
AP	alkaline phosphatase
AST	aspartate aminotransferase
BMI	body mass index
CAP	controlled attenuation parameter
DCP	des-gamma-carboxyprothrombin
ER	endoplasmic reticulum
FGF19/21	fibroblast growth factor 19/21
γGT	gamma-glutamyltransferase
GLDH	glutamate dehydrogenase
GLP1	glucagon-like peptide
HCC	hepatocellular carcinoma
HDL	high-density lipoprotein
IL6	interleukin 6
LBP1	lipoprotein-binding protein 1
LDH	lactate dehydrogenase
LDL	low-density lipoprotein
NAFLD	non-alcoholic fatty liver disease
NASH	non-alcoholic steatohepatitis
PCA	principal component analysis
ROC	receiver operating characteristic
#SpecAb	spectral abundance
TAG	triglyceride
TE	transient elastography
TGFβ	tumor growth factor beta
TNFα	tumor necrosis factor alpha
