# Supplementary material for: Discovering Biomarkers for Non-Alcoholic Steatohepatitis Patients with and without Hepatocellular Carcinoma Using Fecal Metaproteomics
Source: Int J Mol Sci. 2022 Aug 9;23(16):8841. doi: 10.3390/ijms23168841 (PMC9408600; doi:10.3390/ijms23168841)
Supplement: Supplementary file 1 [file ijms-23-08841-s001.zip › Supplementary Figure S1.pdf]

Supplementary Figures to the manuscript

Discovering biomarkers for non-alcoholic steatohepatitis patients with and without hepatocellular carcinoma using fecal metaproteomics

Svenja Sydor[1], Christian Dandyk[2], Johannes Schwerdt[3], Paul Manka[1], Dirk Benndorf[*2,4], Theresa Lehmann[2], Kay Schallert[2], Udo Reichl[2,4], Ali Canbay[1], Lars P. Bechmann[1], Robert Heyer[2,4,5,6*]

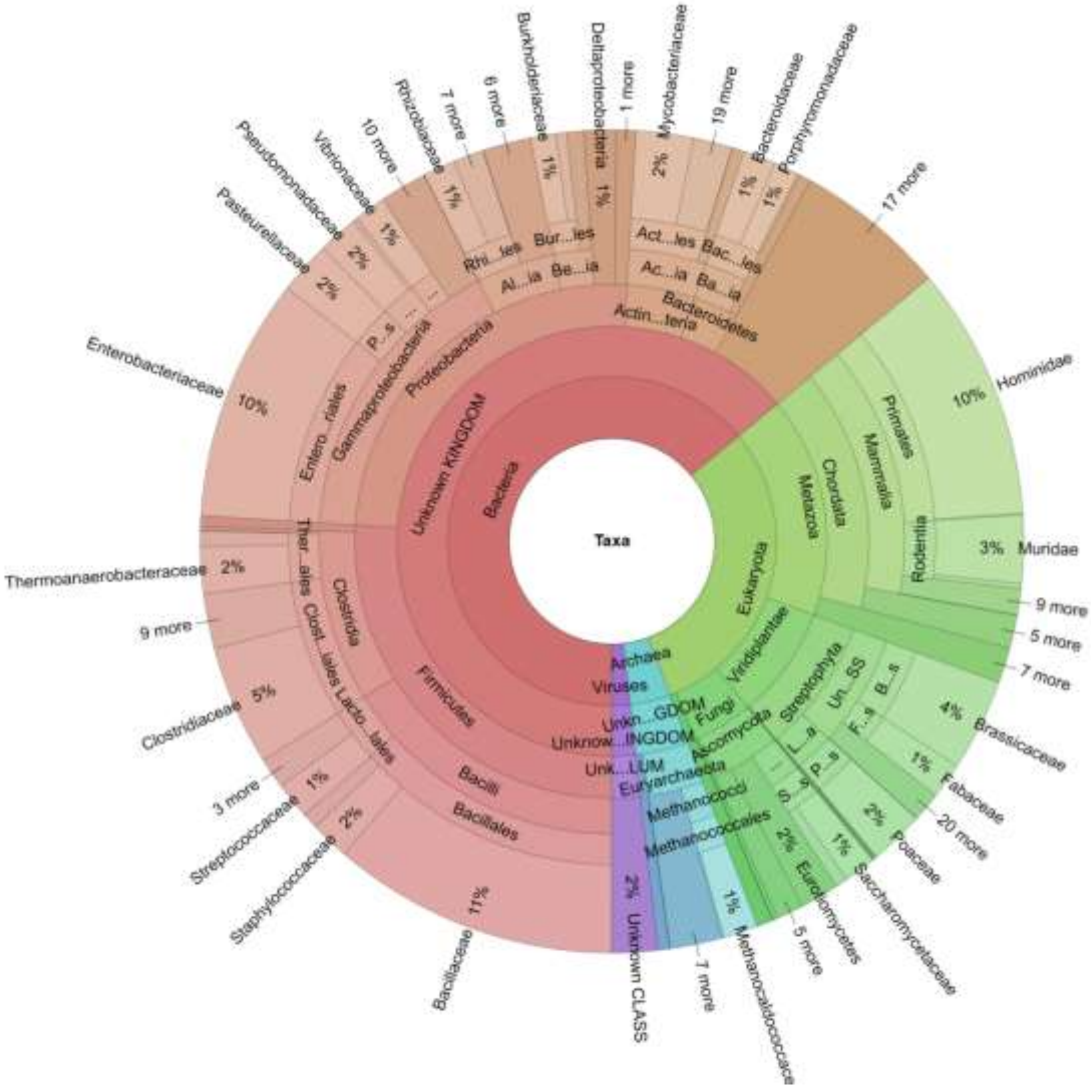

Supplementary Figure S1. Taxonomic assignment of all identified spectra of all patients' fecal samples.

Spectra that could not be linked to a certain superkingdom (30.0% of all identified spectra) or not-annotated metagenome entries (36.5% of all identified spectra) were excluded from the Krona plot.
