# Supplementary material for: Discovering Biomarkers for Non-Alcoholic Steatohepatitis Patients with and without Hepatocellular Carcinoma Using Fecal Metaproteomics
Source: Int J Mol Sci. 2022 Aug 9;23(16):8841. doi: 10.3390/ijms23168841 (PMC9408600; doi:10.3390/ijms23168841)
Supplement: Supplementary file 1 [file ijms-23-08841-s001.zip › Supplementary Table S1.pdf]

Supplementary Table S1. Patient demographics, parameters of transient elastography and steatosis (CAP), serum tumor markers, and serum parameters of liver injury, cell death, inflammation bile acid, lipid and glucose metabolism. Individual p-values are stated in case of significant differences and were calculated by Kruskal-Wallis test. For the single parameters the mean and the standard deviation over all patients is shown.

	Control (C)	NASH (N)	HCC	p-value
**Number of subjects**	m=12/f=7	m=20/f=12	m=22/f=7	
**Age (years)**	23.5 ± 2.7	53.3 ± 14.0	67.8 ± 10.4	<0.001 C vs. N<br><0.001 C vs. HCC<br>0.001 N vs. HCC
**BMI (kg/m²)**	23.0 ± 2.6	30.9 ± 6.5	30.3 ± 5.6	<0.001 C vs. N<br><0.001 C vs. HCC
**TE (kPa)**	5.4 ± 2.4	15.9 ± 14.2	37.1 ± 27.0	<0.001 C vs. N<br><0.001 C vs. HCC<br>0.013 N vs. HCC
**CAP (dB/m)**	209.1 ± 48.0	303.1 ± 71.7	290.1 ± 67.6	<0.001 C vs. N<br>0.003 C vs. HCC
**AFP-Centaur (IU/ml)**	1.0 ± 0.0	36.3 ± 187.8	1234.8 ± 3832.9	<0.001 C vs. N<br><0.001 C vs. HCC<br><0.001 N vs. HCC
**AFP-L3 (%)**	0.3 ± 0.0	8.4 ± 21.6	21.4 ± 25.2	<0.001 C vs. HCC<br>0.002 N vs. HCC
**DCP (ng/ml)**	0.2 ± 0.0	1.7 ± 4.5	157.3 ± 353.9	0.003 C vs. N<br><0.001 C vs. HCC
**AST (U/l)**	26.0 ± 13.7	56.1 ± 43.7	82.7 ± 92.7	0.001 C vs. N<br><0.001 C vs. HCC<br>0.043 N vs. HCC
**ALT (U/l)**	25.4 ± 15.0	66.4 ± 56.6	48.1 ± 19.2	<0.001 C vs. N<br><0.001 C vs. HCC
**AP (U/l)**	56.4 ± 16.5	119.3 ± 71.9	223.8 ± 283.2	<0.001 C vs. N<br><0.001 C vs. HCC<br>0.042 N vs. HCC
**γGT /U/l)**	18.2 ± 8.0	132.6 ± 120.3	235.2 ± 160.7	<0.001 C vs. N<br><0.001 C vs. HCC<br>0.018 N vs. HCC
**GLDH (U/l)**	2.6 ± 0.9	15.6 ± 30.3	12.0 ± 9.9	<0.001 C vs. N<br><0.001 C vs. HCC
**LDH (U/l)**	182.1 ± 38.6	212.1 ± 46.8	251.1 ± 65.1	0.029 C vs. N<br><0.001 C vs. HCC<br>0.036 N vs. HCC
**Bilirubin (mg/dl)**	0.7 ± 0.3	0.7 ± 0.3	1.3 ± 1.2	n. s.
**M65 (U/l)**	142.6 ± 11.2	507.1 ± 417.4	438.7 ± 506.0	<0.001 C vs. N<br><0.001 C vs. HCC
**M30 (U/l)**	116.8 ± 34.8	263.6 ± 200.8	438.7 ± 291.4	0.001 C vs. N<br><0.001 C vs. HCC<br>0.016 N vs. HCC
**IL6 (pg/ml)**	3.1 ± 0.0	9.2 ± 11.7	20.5 ± 59.7	0.001 C vs. N<br>0.001 C vs. HCC
**TNFα (pg/ml)**	18.7 ± 3.6	17.0 ± 1.7	20.5 ± 59.7	<0.001 C vs. HCC<br><0.001 N vs. HCC

**LBP1 (ng/ml)**	184.8 ± 75.7	205.7 ± 79.1	184.4 ± 81.8	n.s.
**Total bile acids (µmol/l)**	4.0 ± 1.4	21.7 ± 4.5	44.5 ± 57.1	0.005 C vs. N<br><0.001 C vs. HCC<br>0.024 N vs. HCC
**FGF19 (pg/ml)**	26.0 ± 13.7	56.1 ± 43.7	82.7 ± 92.7	0.008 N vs. HCC
**TAG (mg/dl)**	113.2 ± 47.2	232.1 ± 145.2	185.2 ± 103.1	0.001 C vs. N<br>0.022 C vs. HCC
**Cholesterol (mg/dl)**	171.8 ± 24.6	204.1 ± 71.9	190.3 ± 33.4	n. s.
**LDL-Cholesterol (mg/dl)**	79.8 ± 15.8	116.5 ± 4.9	115.5 ± 32.9	0.001 C vs. HCC
**HDL-Cholesterol (mg/dl)**	69.4 ± 19.7	48.8 ± 17.9	43.7 ± 18.9	0.001 C vs. N<br><0.001 C vs. HCC
**Adiponectin (ng/ml)**	6.1 ± 3.1	11.3 ± 24.1	13.3 ± 21.9	n. s.
**GLP1 (pg/ml)**	25.4 ± 15.0	66.4 ± 56.6	48.1 ± 19.2	0.001 C vs. N<br><0.001 C vs. HCC
**FGF21 (pg/ml)**	56.4 ± 16.5	119.3 ± 71.9	223.8 ± 283.2	0.001 C vs. N<br>0.001 C vs. HCC
